# Ventricular assist device support in neonates and infants with a failing functionally univentricular circulation

**DOI:** 10.1016/j.xjtc.2021.09.056

**Published:** 2021-10-19

**Authors:** Mark S. Bleiweis, James C. Fudge, Giles J. Peek, Himesh V. Vyas, Susana Cruz Beltran, Andrew D. Pitkin, Kevin J. Sullivan, Jose F. Hernandez-Rivera, Joseph Philip, Jeffrey P. Jacobs

**Affiliations:** a Division of Cardiovascular Surgery, Departments of Surgery and Pediatrics, Congenital Heart Center, University of Florida, Gainesville, Fla; b Division of Pediatric Cardiology, Department of Pediatrics, Congenital Heart Center, University of Florida, Gainesville, Fla; c Department of Anesthesia, Congenital Heart Center, University of Florida, Gainesville, Fla; d Division of Pediatric Critical Care, Department of Pediatrics, Congenital Heart Center, University of Florida, Gainesville, Fla

**Keywords:** ventricular assist device, functionally univentricular heart, hypoplastic left heart syndrome, hypoplastic right heart syndrome

## Abstract

Some neonates with functionally univentricular hearts are at extremely high risk for conventional surgical palliation. Primary cardiac transplantation offers the best option for survival of these challenging neonates; however, waitlist mortality must be minimized. We have developed a comprehensive strategy for the management of neonates with functionally univentricular hearts that includes the selective use of conventional neonatal palliation in standard-risk neonates, hybrid approaches in neonates with elevated risk secondary to a noncardiac etiology, and neonatal palliation combined with insertion of a single ventricular assist device (VAD) in neonates with elevated risk secondary to a cardiac etiology. Here we describe our selection criteria, technical details, management strategies, pitfalls, and current outcomes for neonates with functionally univentricular hearts supported with a VAD. Our experience shows that extremely high-risk neonates with functionally univentricular hearts who are poor candidates for conventional palliation can be successfully stabilized with concomitant palliation and pulsatile VAD insertion while awaiting cardiac transplantation.

**Figure:**
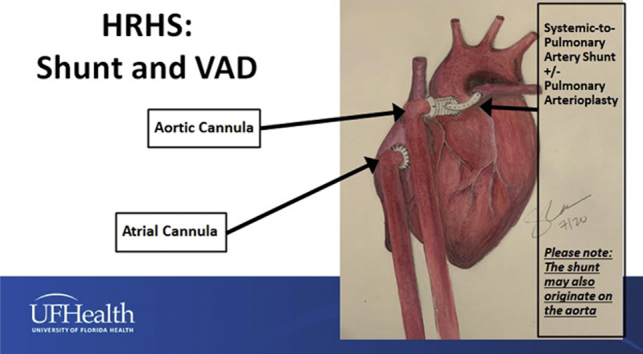
Palliation plus VAD for HRHS with a VAD and a systemic-to-pulmonary shunt with or without pulmonary arterioplasty.

**Central Message**

Extremely high-risk neonates with functionally univentricular hearts who are poor candidates for conventional palliation can be successfully stabilized with a ventricular assist device while awaiting cardiac transplantation.

See Commentaries on pages 205 and 207.

The survival of patients with congenital heart disease continues to improve after surgical palliation and repair; however, certain subsets of patients remain at high risk. We have developed a comprehensive strategy for the management of neonates and infants with functionally univentricular hearts that includes the selective use of conventional neonatal palliation in standard-risk patients, hybrid approaches in neonates with elevated risk secondary to noncardiac etiology, and neonatal palliation combined with insertion of a single ventricular assist device (VAD) in neonates with elevated risk secondary to cardiac etiology (Figures 1 and 2).^1^, ^2^, ^3^, ^4^, ^5^ Our unique strategy has resulted in improved survival of children with functionally univentricular circulation. In this report, we describe our selection criteria, technical details, management strategies, pitfalls, and current outcomes for neonates with functionally univentricular hearts supported with VAD.

**Figure 1:**
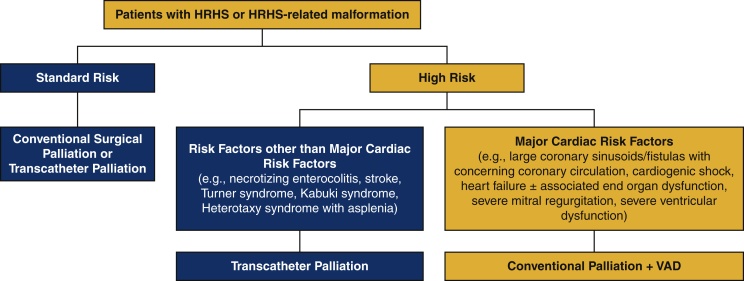
Comprehensive approach to patients with hypoplastic right heart syndrome (*HRHS*) showing our pathway for decision making in neonates with HRHS. The pathway for the patients who are candidates for Palliation + VAD is shown in the *orange boxes*. *VAD*, Ventricular assist device.

**Figure 2:**
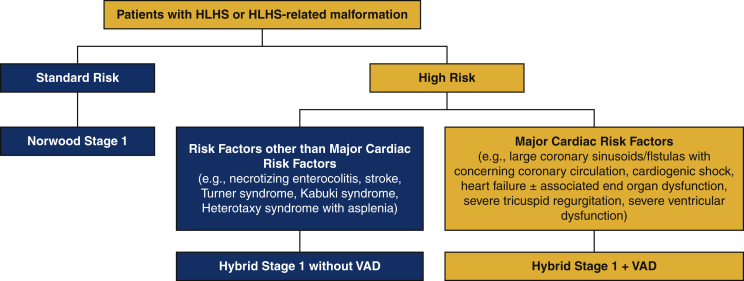
Comprehensive approach to patients with hypoplastic left heart syndrome (*HLHS*), showing our pathway for decision making in neonates with HLHS. The pathway for the patients who are candidates for Hybrid + VAD is shown in the *orange boxes*. *VAD*, Ventricular assist device.

## Selection Criteria

We have developed a comprehensive strategy for the management of neonates with functionally univentricular hearts, including neonates with both hypoplastic right heart syndrome (HRHS) and hypoplastic left heart syndrome (HLHS).

For neonates with HRHS (Figure 1):

• Standard-risk patients undergo initial palliation with either a surgical or transcatheter approach.
• Neonates with risk factors other than major cardiac risk factors (eg, clinically significant necrotizing enterocolitis, stroke prior to initial surgical palliation, Heterotaxy syndrome with asplenia, congenital third-degree atrioventricular block, and/or genetic syndromes such as Turner syndrome [i.e., chromosomal karyotype 45X0]^5^ and Kabuki syndrome) undergo initial transcatheter palliation.
• Neonates with elevated risk secondary to cardiac etiology (eg, large coronary sinusoids/fistulas with concerning coronary circulation, cardiogenic shock, heart failure with or without associated end-organ dysfunction, severe mitral regurgitation, and/or severe ventricular dysfunction) undergo initial VAD insertion (EXCOR; Berlin Heart, Berlin, Germany) along with initial surgical palliation (Palliation + VAD). The pathway for the patients who are candidates for Palliation + VAD is shown in the orange boxes in Figure 1.

For neonates with HLHS or HLHS-related malformations (Figure 2):

• Standard-risk patients undergo initial palliation with the Norwood (Stage 1) Operation, typically with a right ventricle–to–pulmonary artery conduit. Neonates undergoing Norwood (Stage 1) Operation with a dominant morphologic left ventricle (eg, tricuspid atresia with transposed great arteries or double-inlet left ventricle with transposed great arteries) will undergo placement of a systemic–to–pulmonary artery shunt as their source of pulmonary blood flow.
• Neonates with risk factors other than major cardiac risk factors (eg, clinically significant necrotizing enterocolitis, stroke prior to initial surgical palliation, Heterotaxy syndrome with asplenia, congenital third-degree atrioventricular block, and/or genetic syndromes such as Turner syndrome [i.e., chromosomal karyotype 45X0]^5^ and Kabuki syndrome) undergo initial Stage 1 Hybrid Palliation consisting of application of bilateral pulmonary artery bands, stent placement in the patent arterial duct, and atrial septostomy if needed.
• Neonates with elevated risk secondary to cardiac etiology (eg, large coronary sinusoids/fistulas with concerning coronary circulation, cardiogenic shock, heart failure with or without associated end-organ dysfunction, severe tricuspid regurgitation, and/or severe ventricular dysfunction) undergo initial VAD insertion (Berlin EXCOR; Berlin Heart, Berlin, Germany) along with initial Stage 1 Hybrid Palliation consisting of application of bilateral pulmonary artery bands, stent placement in the patent arterial duct, and atrial septectomy if needed (Hybrid + VAD). The pathway for the patients who are candidates for Hybrid + VAD is shown in the orange boxes in Figure 2.

Of note, it is possible to place bilateral pulmonary artery bands and maintain ductal patency with prostaglandin in neonates with HLHS (or HLHS-related malformations) and elevated risk secondary to noncardiac etiology, to allow the patient time to recover before undergoing the Norwood (Stage 1) Operation after a few weeks on prostaglandin. This approach allows resuscitation of the high-risk neonate with pulmonary overcirculation in preparation for a Norwood (Stage 1) Operation during the same hospitalization. We have not used this approach and prefer to place a ductal stent in these patients in preparation for a Comprehensive Stage 2 Operation.

## Technical Details

### Surgical Technique for Hybrid + VAD for HLHS

After a median sternotomy and pericardiotomy are performed and the cardiac structures are evaluated, the subrectus space, which will be used to tunnel the Berlin cannulae, is created by dissecting the peritoneum off of the posterior aspect of the rectus muscles. The 6-mm VAD inflow cannula can be tunneled at this time, exiting on the right side, clear of the costal margin. The right-sided incision is placed so that the cannula will sit at the correct location in the right atrium, and the Teflon felt on the VAD inflow cannula is ultimately positioned at approximately its midpoint with respect to the skin incision. In a neonate or small infant, the exit site is often at or below the umbilicus to ensure adequate Teflon below the skin for incorporation. The tunnel is dilated serially up to the size of a 9-mm dilator, and the canula is then passed through the tunnel. Then the cannula tip is parked in the right pleural space for later insertion and use.

The patient is systemically heparinized. In preparation for cardiopulmonary bypass (CPB), cannulation is performed in the patent arterial duct (and/or the innominate artery), superior vena cava, and inferior vena cava. The right and left pulmonary arteries are banded using a cut ring of a 4-mm polytetrafluoroethylene (PTFE) graft. The bands are sized to approximately 3.75 mm to allow room for growth while on the waitlist. (A 3.5-mm PTFE graft was previously used and still is used occasionally in smaller patients.) A hemoclip is placed as a radiographic marker at the junction of the left pulmonary artery and the arterial duct to aid subsequent positioning of the ductal stent.

The right atrium is prepared with 2 pledgeted 5-0 polypropylene (Prolene; Ethicon, Somerville, NJ; https://www.jnjmedicaldevices.com/en-US/companies/ethicon) purse string sutures, with the pledgets positioned at the cranial and caudal ends of the planned VAD inflow cannulation site. This VAD inflow cannulation site is positioned at the convexity of the right atrial free wall slightly inferior to the atrial appendage in a position so as to allow positioning of the VAD inflow cannula in the mid-right atrial cavity facing posteromedially at an angle of approximately 45 degrees. CPB is then established, and the patent arterial duct is snared with a silk ligature, incorporating the arterial CPB perfusion canula. Caval snares are applied. The atrium is then opened, and an atrial septectomy is performed if needed.

The previously placed 5-0 polypropylene purse string sutures are passed through the cuff of the VAD inflow cannula, 180 degrees from one another. The VAD inflow cannula is inserted into the atrium through the atriotomy incision purse strings, the needles of the purse string sutures are kept in place, and the purse strings are tied. The VAD inflow cannula is then secured by anastomosing the atrial wall to the polyurethane cuff of the VAD inflow cannula, using a continuous running suture with the same previously placed 5-0 polypropylene atrial purse string suture. The VAD inflow cannula is connected to a pump sucker using a ¼-inch connecter to vent the common atrium.

The 5- to 6-mm outflow cannula of the 10-mL Berlin EXCOR VAD is prepared by attaching an 8-mm Dacron graft extension to the end of the outflow cannula, using 4 cardinally placed 5-0 polypropylene simple sutures, so that the cannula tip and the Dacron graft extension are orthogonal to the main body of the outflow cannula. The graft is then secured by tying two #1 silk ligatures to engage the flange on the cannula. The main pulmonary artery is opened longitudinally, and the arteriotomy is enlarged slightly with a punch. The graft is then cut to length (usually at least 10 mm), beveled at an angle optimal to flow, and anastomosed to the main pulmonary artery with 5-0 polypropylene. We make the outflow graft extension of the cannula at least 1 cm so that we have the flexibility of placing the outflow canula in an optimal position within the chest. The medial skin incision is placed so that the VAD outflow cannula will be in the correct location in the chest, and the VAD outflow canula is also sized to ensure that the Teflon felt on the cannula is positioned at approximately its midpoint with respect to the skin incision. The tunnel through the previously created subrectus plane is dilated serially up to the size of a 9-mm dilator, and the VAD outflow cannula is then passed through the tunnel and the medial skin incision. The Berlin Heart cannulas are connected to the VAD after adequate deairing, and the transition is made from CPB to VAD. Heparin is reversed, and the cannulas for CPB are removed.

As discussed below, this procedure can be safely performed in a hybrid operating suite or sequentially by starting in the operating theater for the portion performed on CPB with subsequent intraoperative transfer to the cardiac catheterization laboratory for ductal stent placement under fluoroscopy. Under fluoroscopy, the right ventricle is cannulated via the modified Seldinger technique using a 21-gauge 2.5-cm Cook One-Part Percutaneous Entry Needle (Cook Medical, Bloomington, Ind). A 0.018 in × 40 cm Nitinol Mandrel guidewire (B. Braun Interventional Systems, Bethlehem, Pa) is advanced through the needle into the main pulmonary artery and across the arterial duct. The needle is exchanged for with a 4F Micro-Introducer (B. Braun Interventional Systems), followed by exchange of the wire for a 0.035 in × 180 cm Magic Torque guidewire (Boston Scientific, Marlborough, Mass) that is advanced into the descending aorta. The Micro-Introducer is removed and exchanged for a 7F 5.5-cm Cordis BRITE TIP Interventional Sheath Introducer (Cardinal Health, Santa Clara, Calif). Angiography of the arterial duct is performed through the sheath and used to obtained measurements of the arterial duct for stenting. The arterial duct is visualized by contrast angiography on the lateral plane. The stent is typically 1 to 2 mm larger than the size of the descending thoracic aorta at the aortic isthmus and must be long enough to cover the entire span of the ducal tissue. The proximal end of the ductal stent is positioned at the previously placed hemoclip that is located at the junction of the left pulmonary artery and the arterial duct, with the distal end of the ductal stent extending just slightly past the junction of the aortic arch onto the arterial duct.

**Figure 3:**
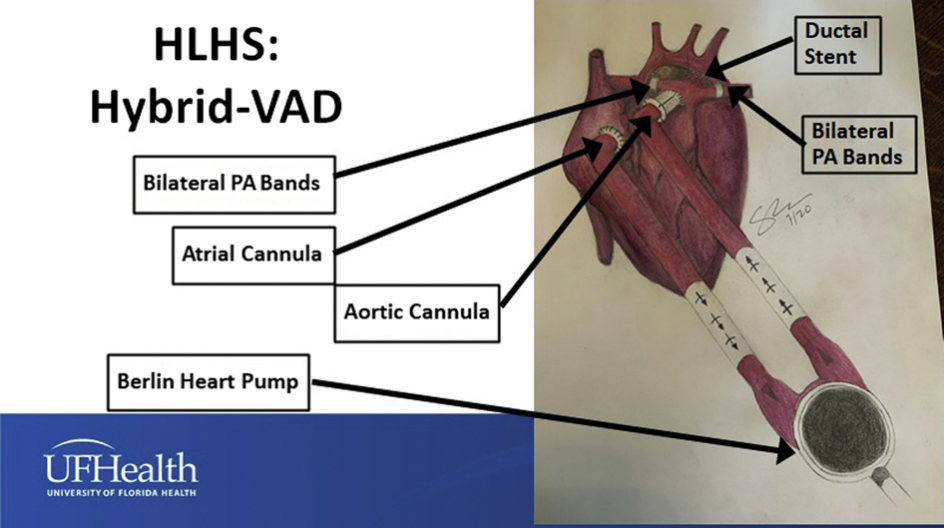
Configuration of Hybrid + VAD for hypoplastic left heart syndrome using application of bilateral pulmonary artery bands, stent placement in the patent arterial duct, atrial septectomy if needed, and Berlin Heart VAD insertion. *VAD*, Ventricular assist device; *PA*, pulmonary artery.

An appropriately sized Cook Zilver 635 Vascular Self-Expanding Stent (Cook Medical) is then deployed in the arterial duct. This stent is typically 7 to 9 mm in diameter and 20 mm long. Postprocedure angiography is performed to confirm appropriate stent position, assess retrograde arch flow, and confirm adequate pulmonary artery banding. The sheath in the right ventricle is removed, and hemostasis is achieved. Usually, delayed sternal closure is performed on the first day after VAD insertion. Figure 3 demonstrates the surgical strategy and shows the configuration of the Hybrid + VAD for HLHS.

### Surgical Technique for Palliation + VAD for HRHS

The technique for HRHS is identical to that used for HLHS, with the following notable differences:

• The aorta, rather than the innominate artery or arterial duct, is used for arterial cannulation for CPB.
• The aorta is used for the VAD outflow cannula.
• A source of pulmonary blood flow is established with either ductal stenting or the creation of a systemic–to–pulmonary artery shunt with or without pulmonary arterioplasty.
• The systemic–to–pulmonary artery shunt can originate from the ascending aorta or from the Dacron extension of the VAD outflow cannula, depending on the aortic dimensions and the surrounding anatomy. This decision is made to prevent kinking of the shunt or compression of the shunt by the VAD outflow cannula, especially at the time of sternal closure.

**Figure 4:**
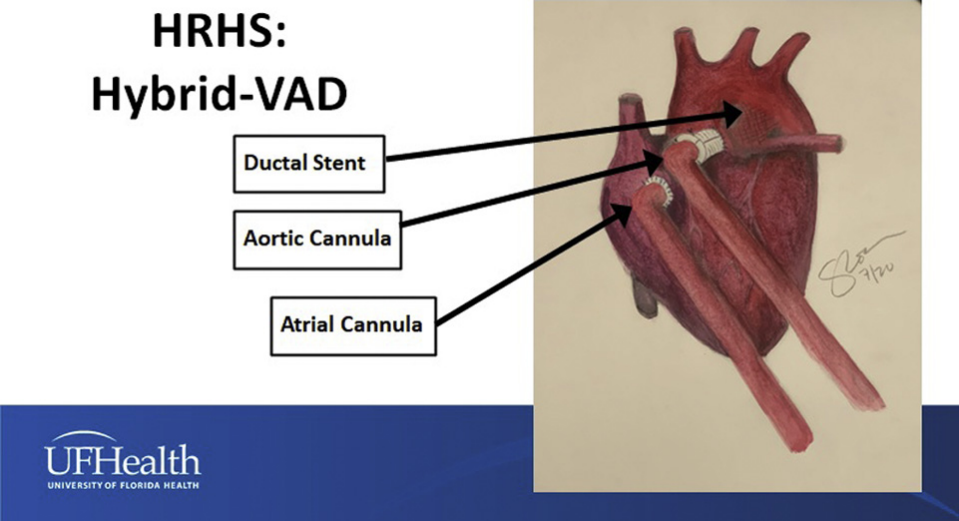
Configuration of Palliation + VAD for hypoplastic right heart syndrome using stent placement in the patent arterial duct, atrial septectomy if needed, and Berlin Heart VAD insertion. *VAD*, Ventricular assist device.

**Figure 5:**
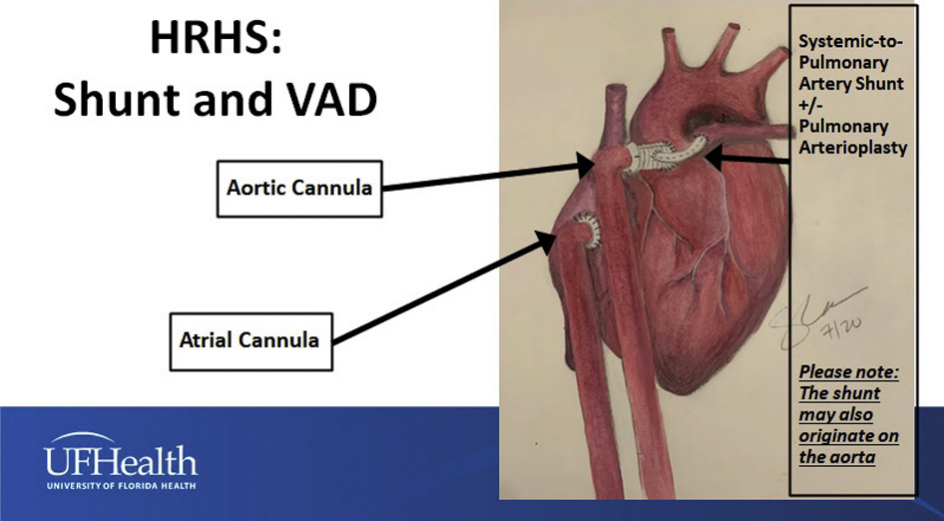
Configuration of Palliation + VAD for hypoplastic right heart syndrome using a systemic-to-pulmonary artery shunt with or without pulmonary arterioplasty, atrial septectomy if needed, and Berlin Heart VAD insertion. The systemic-to-pulmonary artery shunt may originate from the 8-mm Dacron graft extension connecting the outflow cannula to the aorta (as shown in this drawing) or from the aorta itself. *VAD*, Ventricular assist device.

Figures 4 and 5 demonstrate the surgical strategy and show the configurations of Palliation + VAD for HRHS with ductal stenting (Figure 4) and Palliation + VAD for HRHS with the creation of a systemic–to–pulmonary artery shunt with or without pulmonary arterioplasty (Figure 5).

### VAD Logistics: Location of the Procedure and Composition of the TEAM

Insertion of a VAD in the neonate or infant can be safely performed in a hybrid operating suite, or sequentially starting in the operating theater for the portion performed on CPB, followed by subsequent intraoperative transfer to the cardiac catheterization laboratory for ductal stent placement under fluoroscopy. Management of the neonate and infant supported with a VAD requires excellence from all members of the multidisciplinary team.

### VAD Management

The VAD settings are titrated to achieve an initial cardiac index of 4 L/min/m^2^. Target hemodynamic and physiologic parameters are similar to those of any other Stage 1 procedure. The VAD can be upsized from a 10-mL Berlin EXCOR VAD to a 15-mL Berlin EXCOR VAD if necessary secondary to growth of the patient while on the waitlist.

Bivalirudin is initiated on postoperative day 1. The VAD rate is gradually increased as needed to assure adequate cardiac output and systemic tissue perfusion. The patient is extubated as soon as possible. Appropriate weight gain and end-organ function are maintained on VAD support until transplantation.

### Anticoagulation

During the first 24 hours after VAD insertion, no anticoagulation is given. The following anticoagulation protocol is then initiated:

• Bivalirudin: During hours 24 to 72, bivalirudin is titrated to a partial thromboplastin time (PTT) of 50 to 70. After 72 hours, bivalirudin is titrated to a PTT of 70 to 100.
• Aspirin: For patients with a systemic-to-pulmonary artery shunt, aspirin is started on the initial night of VAD insertion at a dose of 5 milligrams/kilogram/day (divided into two daily doses), and aspirin is increased each week until a dose of 30 milligrams/kilogram/day is reached by week 4. For patients without a systemic-to-pulmonary artery shunt, aspirin is started on day 5 after VAD implantation at a dose of 5 mg/kg/day (divided into 2 daily doses), and aspirin is increased each week until a dose of 30 mg/kg/day is reached by week 4.
• Dipyridamole: Dipyridamole is started on week 5 after VAD implantation at a dose of 2.5 mg/kg/day, and dipyridamole is increased twice each week until a dose of 15 mg/kg/day is reached by week 6.
• Omega-3 fatty acid: Omega-3 fatty acid is typically started at 3 to 4 months after VAD implantation.

## Potential Pitfalls: Stroke While on VAD

In this analysis, a stroke is defined as any confirmed neurologic deficit of abrupt onset caused by a disturbance in blood flow to the brain, when the neurologic deficit does not resolve within 24 hours and is associated with radiographic confirmation by computed tomography (CT) scan. In our HLHS cohort, only 2 of 9 patients supported with Hybrid + VAD experienced stroke, and both of these strokes occurred after 150 days on VAD. In our HRHS cohort, 2 of 6 patients supported with Palliation + VAD experienced stroke, one after 90 days on VAD and the other after 10 days on VAD in a patient with Factor V Leiden mutation in whom anticoagulation was stopped secondary to bleeding from the VAD cannula.

None of these strokes were life-threatening or necessitated VAD removal. Although we did not use clopidogrel in these 15 patients, our program is considering augmenting our current protocol of anticoagulation while on VAD, including possibly adding clopidogrel after 120 days on VAD support. In the 4 patients who experienced stroke, both bivalirudin and dipyridamole were stopped following diagnosis. CT was then repeated 3 days after the stroke, and if no evidence of bleeding or progression of the stroke was documented, bivalirudin was restarted with an initial PTT goal of 50 to 70. CT was repeated again 5 days after the stroke, and if no evidence of bleeding or progression of the stroke was documented, dipyridamole was restarted.

## Current Outcomes

Our current approach is based on the following principles: (1) some patients with HLHS or HRHS are at extremely high risk for conventional surgical palliation; and (2) primary cardiac transplantation offers the best option for survival of these challenging neonates; however, waitlist mortality must be minimized.

We have supported 15 high-risk neonates and infants with HLHS or HRHS using the approach described in this article with Palliation + VAD insertion in preparation for cardiac transplantation. Nine high-risk neonates and infants with HLHS were stabilized with a Hybrid + VAD using a VAD, bilateral PA bands, and a ductal stent with or without atrial septectomy. Six high-risk neonates and infants with HRHS were stabilized with Palliation + VAD using a VAD plus a central shunt or a VAD plus a ductal stent.

The 9 patients with HLHS had the following anatomic and/or physiologic features associated with an increased risk for conventional univentricular palliation:

• 4 patients with unfavorable coronary anatomy (large fistulas) and signs of coronary ischemia
• 3 patients with heart failure (1 patient with heart failure with severe tricuspid regurgitation, 1 late referral with heart failure and associated end-organ dysfunction, and 1 patient with heart failure after a previous hybrid procedure at another institution)
• 2 patients with cardiogenic shock (1 patient with cardiogenic shock needing ECMO and 1 patient with cardiogenic shock and incessant arrhythmia).

The 6 patients with HRHS had the following anatomic and/or physiologic features associated with increased risk for conventional univentricular palliation:

• 3 patients with unfavorable coronary anatomy (large fistulas) and signs of coronary ischemia
• 1 patient with signs of coronary ischemia without obvious fistulas
• 1 patient with cardiogenic shock
• 1 patient with cardiogenic shock and bridge from extracorporeal cardiopulmonary resuscitation (ECPR).

In this analysis, we utilize the definition of Operative Mortality that is utilized by The Society of Thoracic Surgeons (STS). Operative Mortality is defined in all STS databases as “(1) all deaths, regardless of cause, occurring during the hospitalization in which the operation was performed, even if after 30 days (including patients transferred to other acute care facilities); and (2) all deaths, regardless of cause, occurring after discharge from the hospital but before the end of the 30^th^ postoperative day.”

Of 9 high risk neonates and infants with HLHS stabilized with a Hybrid + VAD, 7 were neonates and 2 were infants. Eight patients with HLHS underwent Hybrid + VAD as their initial procedure. One patient with HLHS had undergone bilateral pulmonary artery banding, subtotal atrial septectomy, and ductal stenting at a different institution and underwent VAD placement with completion atrial septectomy and ductal stent revision.

During this same era, at the University of Florida:

• 57 standard-risk patients underwent an initial Norwood (Stage 1) Operation with an Operative Mortality of 2 out of 57 = 3.5%.
• 9 patients with risk factors other than major cardiac risk factors (eg, clinically significant necrotizing enterocolitis [n = 2], stroke prior to initial surgical palliation [n = 1], Heterotaxy syndrome with asplenia [n = 1], congenital third-degree atrioventricular block [n = 1], portal vein thrombosis suggestive of liver infarction [n = 1], and genetic syndromes such as Turner syndrome [i.e., chromosomal karyotype 45X0, n = 2]^5^ and Kabuki syndrome [n=1]) underwent an initial Hybrid Approach “Stage 1” without VAD (8 are alive and 1 died after Comprehensive Stage 2 Operation).
• 3 patients with cardiac risk factors (early in this series) were supported with prostaglandin while awaiting primary cardiac transplantation (these patients would likely be supported with Hybrid + VAD today).

Of 6 high risk neonates and infants with HRHS stabilized with a Palliation + VAD, 4 were neonates and 2 were infants. Five patients with HRHS underwent Palliation + VAD as their initial procedure. One infant with HRHS underwent Palliation + VAD after prior central shunt and prior extracorporeal cardiopulmonary resuscitation and prior extracorporeal membrane oxygenation.

During this same era, at University of Florida, 40 neonates and infants underwent initial conventional surgical palliation for HRHS (24 patients underwent systemic-to-pulmonary artery shunt, 6 patients underwent main pulmonary artery banding, and 10 patients underwent primary superior cavopulmonary connection [Glenn] operation) with Operative Mortality of 2 out of 40 = 5.0%.

The median age of these 15 high risk neonates and infants with HLHS or HRHS at Palliation + VAD was 21 days (range = 4-143 days). The median weight of these 15 high risk neonates and infants with HLHS or HRHS at Palliation + VAD was 3.47 kilograms (range = 2.43-4.68).

Of these 15 high risk neonates and infants with HLHS or HRHS stabilized with Palliation + VAD, eight patients survive (53.3%) and seven patients died (46.7%). Of the eight survivors, 7 survivors are at home doing well after successful cardiac transplantation and 1 survivor is doing well in the ICU on VAD support awaiting transplantation. Of the 7 deaths, 1 occurred after primary graft failure at the time of transplantation after 287 days with a VAD.

Of note, of 11 high risk neonates with HLHS or HRHS stabilized with Palliation + VAD, 7 patients survive (63.6%) and 4 patients died (36.4%). (Two infants with HLHS were managed with Hybrid + VAD and did not survive: one 100-day-old infant underwent VAD insertion and hybrid revision after failed hybrid procedure at another institution and one 143-day-old previously unpalliated infant with end organ dysfunction underwent Hybrid + VAD as bridge to decision. Also, one infant with HRHS did not survive: a 34-day old infant underwent Palliation + VAD after prior central shunt and prior extracorporeal cardiopulmonary resuscitation and prior extracorporeal membrane oxygenation.)

In 14 patients no longer on VAD, the median duration of VAD support was 120 days (range, 30-287 days):

• In 7 survivors no longer on VAD, median duration of VAD support was 162 days (range = 64-196 days).
• In 7 nonsurvivors no longer on VAD, the median duration of VAD support was 98 days (range, 30-287 days).
• Only 2 of 8 survivors (25%) required intubation for longer than 10 days after Palliation +VAD.

## Discussion

Of these 15 high-risk neonates and infants with HLHS or HRHS stabilized with Palliation + VAD, eight patients survive (53.3%) and seven patients died (46.7%). Of note, of 11 high risk neonates with HLHS or HRHS stabilized with Palliation + VAD, 7 patients survive (63.6%) and 4 patients died (36.4%).

Our analysis of 15 patients with HRHS or HLHS supported with Palliation + VAD demonstrates that high-risk patients with functionally univentricular hearts who are suboptimal candidates for conventional palliation can be successfully stabilized with concomitant palliation and pulsatile VAD insertion while awaiting transplantation; these patients may be extubated and optimized for transplantation while on VAD. In patients with functionally univentricular hearts, we prefer pulsatile rather than continuous flow VAD because we believe that (1) pulsatile VAD is more physiologic, (2) the management of patients on pulsatile VAD is more intuitive for the health care team, (3) pulsatile VAD is associated with decreased risk of pulmonary overcirculation, and (4) pulsatile VAD is associated with improved renal function.

Clearly, not enough donor hearts exist to offer transplantation to all patients with HLHS, let alone all patients with functionally univentricular hearts. However, it is reasonable to offer transplantation to patients at high-risk for conventional staged palliation. Unfortunately, because of the shortage of donor organs, time on the waiting list for a heart can be long, leading to increasing concern about the potential for waitlist mortality. Because of these potentially long waiting times, it is also reasonable to stabilize these high-risk patients with functionally univentricular hearts with Palliation + VAD while awaiting transplantation.^1^, ^2^, ^3^ This approach facilitates early extubation and optimization for transplantation while on VAD in this high-risk population.

Survival of patients weighing <5 kg with functionally univentricular physiology supported with VAD is novel.^6^, ^7^, ^8^, ^9^, ^10^, ^11^ In 2008, Pearce and colleagues^6^ reported successful cardiac transplantation after Berlin Heart insertion and bridging in a 15-month-old boy with a functionally univentricular heart (double-outlet right ventricle [S,D,D], mitral valve atresia, D-malposition of the great vessels, status post-pulmonary artery band in infancy) and poor systemic ventricular function, using an aortopulmonary shunt as a supplementary source of pulmonary blood flow. In 2014, Weinstein and colleagues^7^ reported a retrospective review of the EXCOR Investigational Device Exemption study database that included VAD implants under the primary cohort and the compassionate use cohort between May 2007 and December 2011. Twenty-six of 281 patients supported with a VAD in this analysis had univentricular physiology, including 15 with HLHS. Nine patients were supported after neonatal palliative surgery (systemic–to–pulmonary artery shunt or right ventricle–to–pulmonary artery conduit), 12 after superior cavopulmonary connection, and 5 after total cavopulmonary connection. Eight of 9 patients with a VAD implant after neonatal palliation died, all within 3 weeks of implantation (range, 0-17 days). The only survivor in this series after Stage 1 Palliation was a 17-month-old child who underwent takedown of a previous Damus–Kaye–Stansel procedure and previous systemic-to-pulmonary artery shunt, pulmonary artery reconstruction, and insertion of biventricular VADs.^7^

Also in 2014, Conway and colleagues^8^ reported an analysis of all children weighing <10 kg who were enrolled in the sponsor's US regulatory database and supported with the Berlin Heart EXCOR Pediatric VAD as a bridge to transplantation between May 9, 2007, and December 31, 2010. A total of 97 children weighing <10 kg were included, 33 of whom weighed <5 kg. Outcomes were significantly worse for patients weighing <5 kg compared with those weighing 5 to 10 kg. Only 27.3% (9 of 33) of the children weighing <5 kg experienced a successful outcome (defined as successful transplantation or weaning from the VAD with a good neurologic outcome within 30 days of explantation); 63.6% (21 of 33) died, and 9.1% (3 of 33) were weaned unsuccessfully. (Unsuccessful weaning [i.e., weaning failure] was defined as an unacceptable neurological outcome within 30 days of weaning or before hospital discharge, whichever was longer.) These findings were significantly different from those in patients weighing 5 to 10 kg, who had a 71.9% rate of successful outcome (*P* < .001) and a 25% mortality rate.

In 2019, Adachi and colleagues^9^ reported their single-center experience with centrifugal-flow VAD support in children: 40 implantations in 39 patients (28 with cardiomyopathy and 11 with congenital heart disease, including only 3 with univentricular physiology). Of the 3 patients with univentricular physiology, all had had a systemic right ventricle, 2 had Glenn circulation, and 1 had Fontan circulation. All patients with univentricular physiology were managed as outpatients and underwent cardiac transplantation at 5, 7, and 17 months of VAD support.^9^

In 2021, Puri and Adachi^10^ reviewed the published literature in the form of database and registry reports as well as single-center studies to discuss the outcomes of patients with Stage 1 or 2 single ventricle congenital heart disease receiving VAD support. According to the authors, “the outcomes of Stage I and Stage II SV-CHD patients on VAD support from the Pedimacs database are poor, with less than 50% survival on VAD by the 3-month mark in both.”^10^

Also in 2021, the International Society for Heart and Lung Transplantation published a consensus statement for the selection and management of patients with pediatric and congenital heart disease on a VAD.^11^ This consensus statement was endorsed by the American Heart Association and provides the following key points regarding support strategies for functionally univentricular patients on a VAD^11^:

• To support Stage 1 patients with parallel circulations, VAD flows to achieve a higher cardiac index are often required, and a balanced Qp/Qs is crucial.
• In Stage 2 patients, converting to shunted or Fontan physiology at the time of VAD implantation may be considered for improved pulmonary blood flow.
• There is increasing experience and success using durable VADs to support Fontan patients with heart failure due to systemic ventricular dysfunction.

As documented in the peer reviewed literature, the techniques, strategies, and approach reported in our current article are novel, as evidenced by the paucity of published literature on VAD support in neonates and infants with a failing functionally univentricular circulation.^6^, ^7^, ^8^, ^9^, ^10^, ^11^

### Limitations and Future Directions

The major challenge of prolonged VAD support of neonates and infants is the prevention of thromboembolic complications and stroke. Our program is considering augmenting our current protocol of anticoagulation while on VAD, including the possible addition of clopidogrel after 120 days on VAD support.

## Conclusions

Our experience documents that extremely high-risk patients with functionally univentricular hearts who are poor candidates for conventional palliation can be successfully stabilized with concomitant palliation and pulsatile VAD insertion while awaiting transplantation. These patients may be extubated, enterally nourished, and optimized for transplantation while on a VAD. The use of a VAD facilitates survival on the transplantation waitlist during prolonged wait times. VAD support allows survival through potential crises while on the waiting list, including episodes of hemodynamic instability and sepsis. Strategies must be developed to prevent stroke in neonates and infants supported with a VAD for prolonged periods.

This strategy of management described in this review is clearly in evolution. We believe that this approach maximizes the chance of survival of extremely high-risk patients with functionally univentricular hearts who otherwise might die awaiting transplantation.

### Conflict of Interest Statement

The authors reported no conflicts of interest.

The *Journal* policy requires editors and reviewers to disclose conflicts of interest and to decline handling or reviewing manuscripts for which they may have a conflict of interest. The editors and reviewers of this article have no conflicts of interest.

## Figures and Tables

**Table:** 

	**JTCVS:** Hybrid procedure with pulsatile ventricular assist device for hypoplastic left heart syndrome awaiting transplantation. Philip J, Reyes K, Ebraheem M, Gupta D, Fudge JC, Bleiweis MS. *J Thorac Cardiovasc Surg.* 2019;158(2):e59-e61.
	**Commentary:** Filling the gap: Can ventricular assist devices support hearts with a single ventricle? Jean-St-Michel E, Mazwi M, Jeewa A. *J Thorac Cardiovasc Surg*. 2019;158(2):e63-e64.
	**Commentary:** Between the devil and the deep blue sea: A new palliation for high-risk neonates with hypoplastic left heart syndrome. Brown ML, Fynn-Thompson F. *J Thorac Cardiovasc Surg*. 2019;158(2):e65-e66.
	**JTCVS:** Pulsatile ventricular assist device as a bridge to transplant for the early high-risk single-ventricle physiology. Philip J, Powers E, Machado D, Colon DL, Gupta D, Shih R, Ebraheem M, Fudge C, Bleiweis M. *J Thorac Cardiovasc Surg*. 2021;162(2):405-413.e4.
	**Commentary:** How to VAD to avoid BAD in high-risk single ventricle. Lee MM, Honjo O. *J Thorac Cardiovasc Surg*. 2021;162(2):414-415.
	**Commentary:** Promise with pulsatility? Early bridging of high-risk patients with single-ventricle physiology with a ventricular assist device. Perry T, Morales DLS. *J Thorac Cardiovasc Surg*. 2021;162(2):415-416.
	**Commentary:** Ventricular assist device support in an early infancy with single ventricle: Transformation from salvage to elective. Moon J, Adachi I. *J Thorac Cardiovasc Surg*. 2021;162(2):416-417.
	**JTCVS:** Successful cardiac transplant after Berlin Heart bridge in a single ventricle heart: use of aortopulmonary shunt as a supplementary source of pulmonary blood flow. Pearce FB, Kirklin JK, Holman WL, Barrett CS, Romp RL, Lau YR. *J Thorac Cardiovasc Surg*. 2009;137(1):e40-2.
	**JTCVS:** The use of the Berlin Heart EXCOR in patients with functional single ventricle. Weinstein S, Bello R, Pizarro C, Fynn-Thompson F, Kirklin J, Guleserian K, Woods R, Tjossem C, Kroslowitz R, Friedmann P, Jaquiss R. *J Thorac Cardiovasc Surg*. 2014;147(2):697-704; discussion 704-5.
	**JTCVS:** Centrifugal-flow ventricular assist device support in children: A single-center experience. Adachi I, Zea-Vera R, Tunuguntla H, Denfield SW, Elias B, John R, Teruya J, Fraser CD Jr. *J Thorac Cardiovasc Surg*. 2019 Apr;157(4):1609-1617.e2.
	**Commentary:** Patience is a virtue: Recovery is only possible if given a chance to happen, but is this safe? Riggs KW, Morales DLS. *J Thorac Cardiovasc Surg*. 2019;157(4):1618-1619.
	**Pediatric Annual:** Mechanical support for the failing single ventricle at pre-Fontan stage: Current state of the field and future directions. Puri K, Adachi I. *Semin Thorac Cardiovasc Surg Pediatr Card Surg Annu*. 2021;24:10-18.
